# Assessing Physicians’ Knowledge, Attitudes, and Practices Regarding Cervical Cancer Prevention, Diagnosis, and Management in Lahore, Pakistan

**DOI:** 10.3390/healthcare14213613

**Published:** 2026-10-26

**Authors:** Maha Amjad, Adeel Aslam, Anwar Ali, Asma Ghulam Mustafa, Ayesha Iqbal, Muhammad Mamoon Iqbal, Salmeen D. Babelghaith, Naji Alqahtani, Adel Bashatah, Wajid Syed

**Affiliations:** 1 Department of Pharmacy, University of Lahore, Lahore 54000, Pakistan; 2 Department of Pharmacy Practice and Clinical Pharmacy, Faculty of Pharmacy, Universiti Teknologi MARA, Campus Puncak Alam, Puncak Alam 42300, Malaysia; 3 Trauma Center DHQ Hafizabad, Hafizabad 52110, Pakistan; 4 Department of Pharmacy Practice and Policy, University Park Campus, University of Nottingham, Nottingham NG7 2RD, UK; aiqbal6@ualberta.ca; 5 Office of Lifelong Learning and the Physician Learning Program, Faculty of Medicine and Dentistry, University of Alberta, Edmonton, AB T6G 1C9, Canada; 6 Department of Respiratory Sciences, Institute for Lung Health, University of Leicester, Leicester LE1 7RH, UK; 7 Department of Respiratory Medicine, National Institute for Health Research Leicester Biomedical Research Centre—Respiratory, University Hospitals of Leicester NHS Trust, Glenfield Hospital, Leicester LE3 9QP, UK; 8 Department of Clinical Pharmacy, College of Pharmacy, King Saud University, Riyadh 11451, Saudi Arabia; 9 Department of Nursing Administration and Education, College of Nursing, King Saud University, Riyadh 11451, Saudi Arabiaabashatah@ksu.edu.sa (A.B.)

**Keywords:** cervical cancer, knowledge, attitude, practices, Lahore, Pakistan

## Abstract

**Background**: Cervical cancer is a major public health concern in Pakistan, where physicians play an important role in prevention, screening, counseling, and early detection. This study assessed physicians’ knowledge, attitudes, and self-reported practices regarding cervical cancer. **Methods**: This study employed a cross-sectional design and included 408 physicians selected through snowball sampling techniques. A well-designed questionnaire with closed-ended questions was used for data collection. Statistical analysis included descriptive statistics and inferential statistics such as Chi-square, Mann–Whitney, Kruskal–Wallis, Dunn’s tests, Bonferroni adjustment and binary logistic regression were performed using SPSS version 25. **Results**: Among the 408 physicians, most were young (18–33 years), with an MBBS qualification and 1–5 years of experience. Knowledge scores were highest among those aged 26–33 (*p* < 0.001), those with MBBS and FCPS qualifications (*p* = 0.004) and hospital-based healthcare workers (*p* < 0.001). Attitudes were generally positive, with 41.7% agreeing that cervical cancer is highly prevalent and 33.6% prioritizing early detection. Overall, 56.9% of physicians were classified as having good practices. Practices were influenced by education (*p* < 0.001) and healthcare setting (*p* < 0.01) in the adjusted analysis. Barriers identified included limited resources (77.2%), cultural factors (72.3%), and patient awareness (80.4%). **Conclusions**: Physicians demonstrated generally favorable cervical cancer-related knowledge and several preventive practices, but important differences existed across professional and healthcare-setting characteristics. Targeted continuing education, standardized preventive protocols, and improved patient awareness are needed to strengthen consistent cervical cancer prevention and screening practices in Pakistan.

## 1. Introduction

Cervical cancer remains an important global public health problem despite being largely preventable through HPV vaccination, screening, and appropriate treatment of precancerous lesions [1,2]. The burden of cervical cancer continues to rise, with a disparity in incidence and mortality between developed and developing nations [1,3]. Cervical cancer is the most prevalent gynecological malignancy among women worldwide and remains a major public health concern [4]. More than 95% of cervical cancer-related deaths are projected to occur in low- and middle-income countries (LMICs) by 2030, highlighting the disproportionate burden faced by resource-limited nations like Pakistan [5]. According to the International Agency for Research on Cancer (IARC) and the World Health Organization (WHO), cervical cancer was the fourth most commonly occurring cancer and the fourth leading cause of cancer death among women globally in 2022, with approximately 662,000 new cases and 349,000 deaths estimated worldwide [6]. The majority of these deaths (85%) occur in developing countries, underscoring the urgent need for improved preventive measures [7]. Specifically, in Pakistan, cervical cancer is a critical public health crisis, representing the second most common cancer in women, with high morbidity and mortality due to gaps in prevention, screening, and treatment [8]. In Pakistan, IARC’s GLOBOCAN 2022 estimates indicate approximately 4762 new cases and 3069 deaths from cervical cancer, corresponding to an age-standardized incidence rate of approximately 5.4 per 100,000 women and an age-standardized mortality rate of approximately 3.6 per 100,000 women [9]. High-risk HPV (16/18) infects 10–15% of women, yet vaccination is <5% [10]. Similarly, screening is <5%, with over 70% of cases diagnosed late [11]. These disparities demand urgent policy reforms, resource allocation, and awareness campaigns to reduce Pakistan’s cervical cancer burden and meet global targets. The primary causative factor of cervical cancer is persistent infection with high-risk HPV strains, particularly types 16 and 18, which are responsible for approximately 70% of cases [12,13]. Other well-established risk factors include early sexual debut, multiple sexual partners, high parity, prolonged use of oral contraceptives, smoking, and co-infection with HIV [14,15]. Major symptoms of cervical cancer include abnormal vaginal bleeding, foul-smelling vaginal discharge, and postcoital bleeding, although many women remain asymptomatic until the disease reaches an advanced stage [16,17]. Cervical cancer is largely preventable through two key strategies: HPV vaccination as primary prevention and routine screening for precancerous lesions as secondary prevention [18,19]. Precancerous cervical lesions can remain detectable for up to 10 years or more before progressing to invasive cancer, providing a critical window for intervention [20]. Several challenges hinder the effective implementation of cervical cancer prevention programs [21]. These challenges include limited healthcare infrastructure, which delays timely diagnosis and treatment initiation, along with shortages of essential screening and treatment equipment [22,23]. Additionally, the lack of awareness leads to missed opportunities for early detection and intervention, ultimately affecting patient outcomes [24]. Physicians play a crucial role in cervical cancer prevention by promoting HPV vaccination, encouraging routine screening, and educating women about risk factors and early symptoms [25]. Studies assessing knowledge, attitudes, and practices (KAP) among healthcare providers are essential for strengthening cervical cancer prevention programs [26,27]. The level of knowledge among physicians significantly influences their ability to detect and prevent cervical cancer, emphasizing the need for continuous medical education and training [28]. In Pakistan, there is a critical need to assess the KAP of physicians regarding cervical cancer prevention and management. Despite the increasing burden of cervical cancer, limited research has explored physicians’ knowledge, attitudes, and adherence to screening and treatment guidelines [29]. Addressing this gap is essential for reducing the burden of cervical cancer and improving women’s health outcomes. Therefore, this study aims to assess the knowledge, attitudes, and self-reported practices of physicians regarding cervical cancer in Lahore, Pakistan.

## 2. Methods

### 2.1. Study Design, Duration and Setting

A cross-sectional study was conducted among physicians working in Lahore, Pakistan, between 20 February and 20 August 2024. Data were collected using a structured, self-administered questionnaire. The study was conducted in two tertiary-care hospitals in Lahore, Pakistan.

### 2.2. Study Population

The study population comprised physicians who were involved directly or indirectly in patient care and could potentially contribute to cervical cancer prevention, screening, diagnosis, counseling, referral, or management. Eligible participants included physicians holding MBBS (Bachelor of Medicine and Bachelor of Surgery); FCPS (Fellowship of the College of Physicians and Surgeons); MD (Doctor of Medicine); MBBS and FCPS; and MD and FCPS qualifications or equivalent qualifications recognized by the relevant medical authorities. Physicians working in hospitals, clinics, hospice-care facilities, and other healthcare settings were eligible. Allied healthcare professionals, including nurses, pharmacists, and midwives, were excluded because the study specifically focused on physicians. Participants were informed about the purpose of the study and provided written informed consent before participation.

### 2.3. Sampling Technique and Sample Size

Physicians working at the two participating tertiary-care hospitals were initially approached for participation and were also asked to share the questionnaire with eligible physician peers working in clinics, hospice-care facilities, and other healthcare settings. Thus, the two tertiary-care hospitals served as the initial recruitment sites, while healthcare facility type represented the participants’ reported usual workplace. A snowball sampling technique was used to recruit eligible physicians. Because a reliable estimate of the total number of eligible physicians in Lahore was not available, the sample size was estimated using the standard single-population proportion formula [30,31]:

$$n=\frac{{\left(\frac{\mathrm{Za}}{2}\right)}^{2}P\left(1-P\right)}{d^{2}}$$

where *Z* represents the standard normal deviate corresponding to a 95% confidence level (1.96), *P* represents the assumed proportion, and *d* represents the desired precision (0.05). Because a reliable prior estimate of the proportion of physicians with the outcome of interest was not available, a conservative value of *P* = 0.50 was used. This value was selected because it produces the largest required sample size under the single-proportion approach and should not be interpreted as the prevalence of cervical cancer in Pakistan.

$$n=\frac{{\left(1.96\right)}^{2}0.50(1-0.50)}{{\left(0.05\right)}^{2}}$$

*n* = 384.16

The resulting minimum sample size was 384 participants. To allow for potential non-response, 480 physicians were approached, of whom 408 consented to participate and completed the questionnaire.

### 2.4. Questionnaire Development and Pilot Testing

Data were collected using a structured questionnaire developed following a review of the relevant literature and existing instruments addressing cervical cancer knowledge, attitudes, and practices among healthcare professionals [7,32,33].

Before the main survey, the questionnaire was pilot-tested among 20 physicians to assess clarity, comprehensibility, and the appropriateness of the response options. Face validity was assessed by 10 physicians, while content validity was evaluated by a panel of 10 experts with relevant clinical and academic experience, including specialists in oncology and gynecology. The questionnaire was subsequently revised based on the feedback obtained during the pilot and validation process.

### 2.5. Data Collection Tool

The questionnaire (Supplementary File) consisted of 29 closed-ended questions divided into five sections: demographic characteristics (5 questions), knowledge (9 questions), attitudes (6 questions), practices (6 questions), and challenges and barriers (3 questions). Section S1 collected demographic and professional information, including sex, age, educational qualification, years of healthcare experience, and healthcare facility type. Section S2 assessed knowledge of cervical cancer, including risk factors, symptoms, prevention, screening, and related clinical information. Section S3 assessed attitudes toward cervical cancer prevention and screening. Section S4 assessed reported practices, including recommending screening, discussing cervical health, recommending HPV vaccination, documenting screening-related information, and providing patient education. Section S5 assessed perceived barriers to effective cervical cancer prevention and care, including resource limitations, cultural factors, and lack of patient awareness.

The questionnaire was administered in English. Because English was the language used for the original instrument and administration, responses may have been influenced by participants’ level of English-language comprehension.

### 2.6. Statistical Analysis

Data were entered into Microsoft Excel and subsequently analyzed using IBM SPSS Statistics version 25. Data were checked for completeness, consistency, and implausible values before analysis. Descriptive statistics were used to summarize participant characteristics and responses to individual questionnaire items. Categorical variables were presented as frequencies and percentages, while continuous knowledge scores were summarized using means and standard deviations.

Knowledge items were scored by assigning one point to each correct response and zero points to incorrect or uncertain responses. Each knowledge item used a single-best-answer format, in which participants were instructed to select one response that they considered the most appropriate answer [34]. Thus, the knowledge score ranged from 0 to 9, with higher scores indicating greater knowledge. Higher scores represented greater knowledge. The distribution of knowledge scores was assessed using the Shapiro–Wilk test. Because the knowledge scores did not satisfy the assumption of normality, the Mann–Whitney U test was used for comparisons involving two groups and the Kruskal–Wallis H test was used for comparisons involving more than two groups. Where the Kruskal–Wallis test indicated an overall significant difference, pairwise comparisons were performed using Dunn’s post hoc test with Bonferroni adjustment. The practice section consisted of six questions. For each practice item, responses were dichotomized into “good practice” and “poor practice.” Responses of “Very Frequently” and “Frequently” were classified as good practice, whereas “Occasionally,” “Rarely,” and “Never” were classified as poor practice. Each participant consequently received a binary score for each of the six practice items, resulting in a total of 0–6 good-practice responses (0–100%). A 50% cutoff was prespecified to classify overall practice [35]. Participants achieving a mean proportion of ≥50% good-practice responses were classified as having “good practice,” while those with <50% were classified as having “poor practice.”

Associations between categorical demographic variables and practice categories were assessed using the chi-square test. Binary logistic regression was subsequently performed to examine factors independently associated with good practice. Good practice was coded as the outcome category and poor practice as the reference outcome. Categorical predictors were entered using clearly specified reference categories. Unadjusted odds ratios (ORs) and adjusted odds ratios (AORs), together with 95% confidence intervals (CIs) and *p*-values, were reported. Statistical significance was assessed using a two-sided *p*-value of <0.05.

### 2.7. Ethical Considerations

The ethical approval for this study was obtained from the Ethical Committee of the Private tertiary-care hospitals (IREC-2024-04H) and the Ethical Committee of the Public tertiary-care hospital (IRB/2024/1271/SIMS). The study was conducted in accordance with the principles of the Declaration of Helsinki. Written informed consent was obtained from all participants before data collection.

## 3. Results

### 3.1. Reliability and Validity

The internal consistency of the questionnaire domains was assessed using Cronbach’s alpha. The knowledge section demonstrated a Cronbach’s alpha of 0.634, which exceeds 0.6, indicating modest internal consistency. This value was interpreted cautiously and was considered adequate for the exploratory nature of the study [36]. The attitude section demonstrated a higher reliability with a Cronbach’s alpha value of 0.755, suggesting a good level of internal consistency. The practice section had the highest reliability with a Cronbach’s alpha value of 0.801, reflecting excellent internal consistency. The overall Cronbach’s alpha of 0.840 indicated that the questionnaire as a whole has strong internal consistency, meaning the items collectively measure the intended aspects of knowledge, attitude, and practices related to cervical cancer. Content validity was supported by a Content Validity Index of 0.904, while the Face Validity Index was 0.916. These findings indicate that the questionnaire items were considered relevant, understandable, and appropriate by the participating experts and reviewers.

### 3.2. Demographic Characteristics

A total of 408 physicians participated in the study (Table 1). Of these, 213 (52.2%) were female and 195 (47.8%) were male. Most participants were young physicians, with 158 (38.7%) aged 18–25 years and 154 (37.7%) aged 26–33 years. Smaller proportions were aged 34–41 years (10.0%), 42–49 years (8.1%), or 50 years and above (5.4%). Regarding educational qualification, 237 (58.1%) participants held an MBBS degree, 53 (13.0%) held MBBS and FCPS qualifications, 26 (6.4%) held an MD, 27 (6.6%) held MD and FCPS qualifications, and 65 (15.9%) reported other qualifications. More than half of the participants (54.7%) had 1–5 years of healthcare experience, while 24.3% had 5–10 years, 9.8% had 11–20 years, and 11.3% had more than 20 years of experience. Most participants worked in hospitals (71.3%), followed by other healthcare facilities (18.4%), clinics (8.3%), and hospice-care facilities (2.0%).

**Table 1:** Demographic characteristics of physicians.

Demographic Characteristics		Frequency	Percentage (%)
What is your gender?	Male	195	47.8
	Female	213	52.2
What is your Age?	18–25	158	38.7
	26–33	154	37.7
	34–41	41	10.0
	42–49	33	8.1
	50 and above	22	5.4
What is your education?	MBBS	237	58.1
	MBBS and FCPS	53	13.0
	MD	26	6.4
	MD and FCPS	27	6.6
	Other	65	15.9
How many years of experience in healthcare?	1–5 years	223	54.7
	5–10 years	99	24.3
	11–20 years	40	9.8
	More than 20 years	46	11.3
Healthcare Facility Type	Hospital	291	71.3
	Clinic	34	8.3
	Hospice care facility	8	2.0
	Other	75	18.4

MBBS = Bachelor of Medicine and Bachelor of Surgery; FCPS = Fellowship of the College of Physicians and Surgeons; MD = Doctor of Medicine.

### 3.3. Knowledge Scores of Physicians

Table 2 presents the mean knowledge scores for various demographic characteristics. The mean knowledge score among male physicians was 7.50 ± 0.95 compared with 7.61 ± 1.05 among female physicians. This difference was not statistically significant (*p* = 0.141). Knowledge scores differed significantly across age groups (*p* < 0.001). Physicians aged 26–33 years had the highest mean knowledge score (7.90 ± 0.89), followed by those aged 34–41 years (7.48 ± 1.16), 18–25 years (7.35 ± 0.92), 42–49 years (7.33 ± 1.19), and 50 years and above (7.18 ± 1.18).

Knowledge also differed significantly according to educational qualification (*p* = 0.004). Physicians with MBBS and FCPS qualifications had the highest mean score (7.88 ± 0.93), whereas the lowest mean scores were observed among physicians with MD and FCPS qualifications (7.22 ± 1.25) and those in the other-qualification category (7.24 ± 1.04). A significant difference was also observed across categories of professional experience (*p* = 0.010). Physicians with 11–20 years of experience had the highest mean score (7.85 ± 1.05), followed by those with 5–10 years (7.60 ± 1.01), 1–5 years (7.58 ± 0.94), and more than 20 years of experience (7.10 ± 1.15). Knowledge scores differed significantly according to healthcare facility type (*p* < 0.001). Hospital-based physicians had a mean score of 7.72 ± 0.90, compared with 7.70 ± 0.97 among clinic-based physicians, 7.12 ± 0.35 among hospice-care physicians, and 6.90 ± 1.18 among physicians working in other settings.

### 3.4. Post Hoc Analysis of Knowledge

Post hoc analysis using Dunn’s test with Bonferroni adjustment was performed following significant Kruskal–Wallis tests (Table 3). Significant pairwise differences were observed across several demographic variables. Physicians aged 26–33 years had significantly higher knowledge scores than those aged 18–25, 42–49, and 50 years and above, whereas the difference between the 26–33- and 34–41-year groups was not statistically significant. Regarding educational qualification, physicians with MBBS and FCPS qualifications had significantly higher knowledge scores than those with MD and FCPS and other qualifications, while MBBS-qualified physicians also had significantly higher scores than those with MD and FCPS and other qualifications. For professional experience, physicians with 11–20, 5–10, and 1–5 years of experience had significantly higher knowledge scores than those with more than 20 years of experience. No other pairwise differences were statistically significant after Bonferroni adjustment. Regarding healthcare facility type, hospital- and clinic-based physicians had significantly higher knowledge scores than physicians working in hospice-care and other settings, whereas the difference between hospital- and clinic-based physicians was not statistically significant. Gender was not included in the Dunn post hoc analysis because it comprised two groups and was assessed separately using the Mann–Whitney U test.

**Table 3:** Post hoc test.

Variables	Group Comparisons	Bonferroni-Adjusted *p*-Value
Age Groups	26–33 vs. 18–25	<0.001 ***
	26–33 vs. 50+	0.002 **
	26–33 vs. 42–49	0.003 **
	26–33 vs. 34–41	0.112
	18–25 vs. 42–49	0.116
	18–25 vs. 50+	0.154
	34–41 vs. 42–49	0.236
	34–41 vs. 50+	0.332
	42–49 vs. 50+	0.672
Education Levels	MBBS and FCPS vs. MD and FCPS	0.005 **
	MBBS and FCPS vs. Other	0.007 **
	MBBS vs. MD and FCPS	0.012 *
	MBBS vs. Other	0.019 *
	MBBS and FCPS vs. MD	0.211
	MBBS vs. MBBS and FCPS	0.084
Years of Experience	11–20 years vs. >20 years	0.006 **
	5–10 years vs. >20 years	0.009 **
	1–5 years vs. >20 years	0.011 *
	1–5 years vs. 5–10 years	0.861
	1–5 years vs. 11–20 years	0.225
	5–10 years vs. 11–20 years	0.368
Healthcare Facility Type	Hospital vs. Hospice Care	<0.001 ***
	Hospital vs. Other	<0.001 ***
	Clinic vs. Hospice Care	0.004 **
	Clinic vs. Other	0.001 **
	Hospital vs. Clinic	0.852

* *p* < 0.05; ** *p* < 0.01; *** *p* < 0.001. MBBS = Bachelor of Medicine and Bachelor of Surgery; FCPS = Fellowship of the College of Physicians and Surgeons; MD = Doctor of Medicine.

### 3.5. Physicians’ Attitudes Toward Cervical Cancer

The attitude findings are presented in Table 4. The attitude items were designed to assess physicians’ perceptions and attitudes toward cervical cancer prevention and management. Some statements represented potentially favorable or unfavorable attitudes rather than recommended clinical practices; therefore, responses to these items were interpreted as measures of attitude and were not used as direct indicators of guideline adherence. Overall, physicians expressed varying levels of agreement with statements concerning cervical cancer prevalence, risk, prevention, screening, and communication with patients. For the statement that cervical carcinoma is highly prevalent and a leading cause of death among malignancies, 170 (41.7%) participants agreed and 62 (15.2%) strongly agreed. Regarding the possibility of cervical cancer occurring in young women, 142 (34.8%) agreed and 67 (16.4%) strongly agreed. For the statement concerning transmission, 128 (31.4%) participants agreed and 63 (15.4%) strongly agreed that cervical carcinoma cannot be transmitted from one person to another. Because cervical cancer itself is not transmitted between individuals, whereas persistent HPV infection can be sexually transmitted, this item should be interpreted carefully and should not be treated as a direct measure of knowledge of HPV transmission.

Early detection was considered important by most participants, with 137 (33.6%) strongly agreeing and 134 (32.8%) agreeing that early detection of cervical cancer is a priority in patient care. Similarly, 78 (19.1%) strongly agreed and 142 (34.8%) agreed that they routinely ask patients about cervical cancer screening. Regarding communication, 73 (17.9%) strongly agreed and 110 (27.0%) agreed that they felt prepared to talk to patients about cervical cancer, whereas 132 (32.4%) remained neutral. This finding suggests that although physicians generally recognized the importance of prevention and early detection, confidence in discussing cervical cancer with patients may require further strengthening.

### 3.6. Physicians’ Reported Practices

The reported practices of physicians are summarized in Table 5. Overall, 207 (50.7%) physicians reported that they very frequently advised patients to undergo Pap smear testing, while 118 (28.9%) reported doing so frequently. HPV vaccination was recommended very frequently by 78 (19.1%) physicians and frequently by 154 (37.7%). Regarding patient counseling, 109 (26.7%) physicians reported that they very frequently provided information and counseling about cervical health, while 164 (40.2%) did so frequently. Documentation and follow-up of cervical screening were reported as very frequent by 47 (11.5%) physicians and frequent by 117 (28.7%). In relation to providing cervical cancer screening knowledge, 61 (15.0%) physicians reported doing so very frequently and 128 (31.4%) frequently. Although most physicians reported engaging in screening-related practices at least frequently, a considerable proportion reported only occasional, rare, or no engagement in several activities. These findings indicate opportunities to strengthen the consistency of cervical cancer counseling, vaccination recommendations, screening documentation, and follow-up.

### 3.7. Level of Reported Practices According to Demographic Characteristics

Table 6 presents the distribution of good- and poor-practice categories according to participant characteristics. Overall, 232 physicians were classified as having good practices and 176 as having poor practices, based on the prespecified response-level classification. Among male physicians, 122 of 195 (62.6%) were classified as having good practices, compared with 110 of 213 (51.6%) female physicians, and the difference was statistically significant (*p* = 0.026). Practice levels also differed significantly across age groups (*p* < 0.001). The highest proportion of good practices was observed among physicians aged 26–33 years (65.6%), followed by those aged 18–25 years (60.8%) and 34–41 years (58.5%). Educational qualification was significantly associated with practice category (*p* < 0.001). The highest proportion of good practices was observed among physicians with MBBS qualifications (71.3%), followed by those with MBBS and FCPS qualifications (54.7%). Professional experience was also significantly associated with practice category (*p* < 0.001). The highest proportion of good practices was observed among physicians with 1–5 years of experience (65.0%), followed by those with 5–10 years of experience (61.6%). Healthcare facility type was significantly associated with practice category (*p* < 0.001). The highest proportion of good practices was observed among clinic-based physicians, with 23 of 34 (67.6%) classified as having good practices, followed by hospital-based physicians, with 186 of 291 (63.9%), and hospice-care physicians, with 5 of 8 (62.5%).

### 3.8. Regression Analysis of Good Practices Related to Cervical Cancer

Binary logistic regression was performed with good practice (coded as 1) as the modeled event. Table 7 presents the regression analysis, highlighting significant demographic factors influencing practices related to cervical cancer. Males were less likely to have good practices compared to females, although this difference became non-significant after adjusting for other factors (ORa = 0.974, *p* = 0.911). Physicians aged 18–25 showed a lower likelihood of good practices compared to those aged 50 and above, and this difference remained non-significant after adjustment (ORa = 1.432, *p* = 0.642). Physicians with an MBBS degree were significantly less likely to report good practices than those with other qualifications, even after adjusting for other factors (ORa = 0.240, *p* < 0.001). Similarly, physicians with 1–5 years of experience were less likely to engage in good practices, and this difference was non-significant after adjustment (ORa = 0.770, *p* = 0.696). Finally, healthcare facility type had a notable impact on practices. Physicians working in hospitals were significantly less likely to engage in good practices (ORa = 0.376, *p* = 0.012). In contrast, physicians working in clinics and hospice care facilities were less likely to report good practices, with significant *p*-values for clinics (ORa = 0.199, *p* = 0.003) and non-significant *p*-values for hospice care facilities (ORa = 0.228, *p* = 0.088).

### 3.9. Barriers to Cervical Cancer-Related Practices

**Figure 1:**
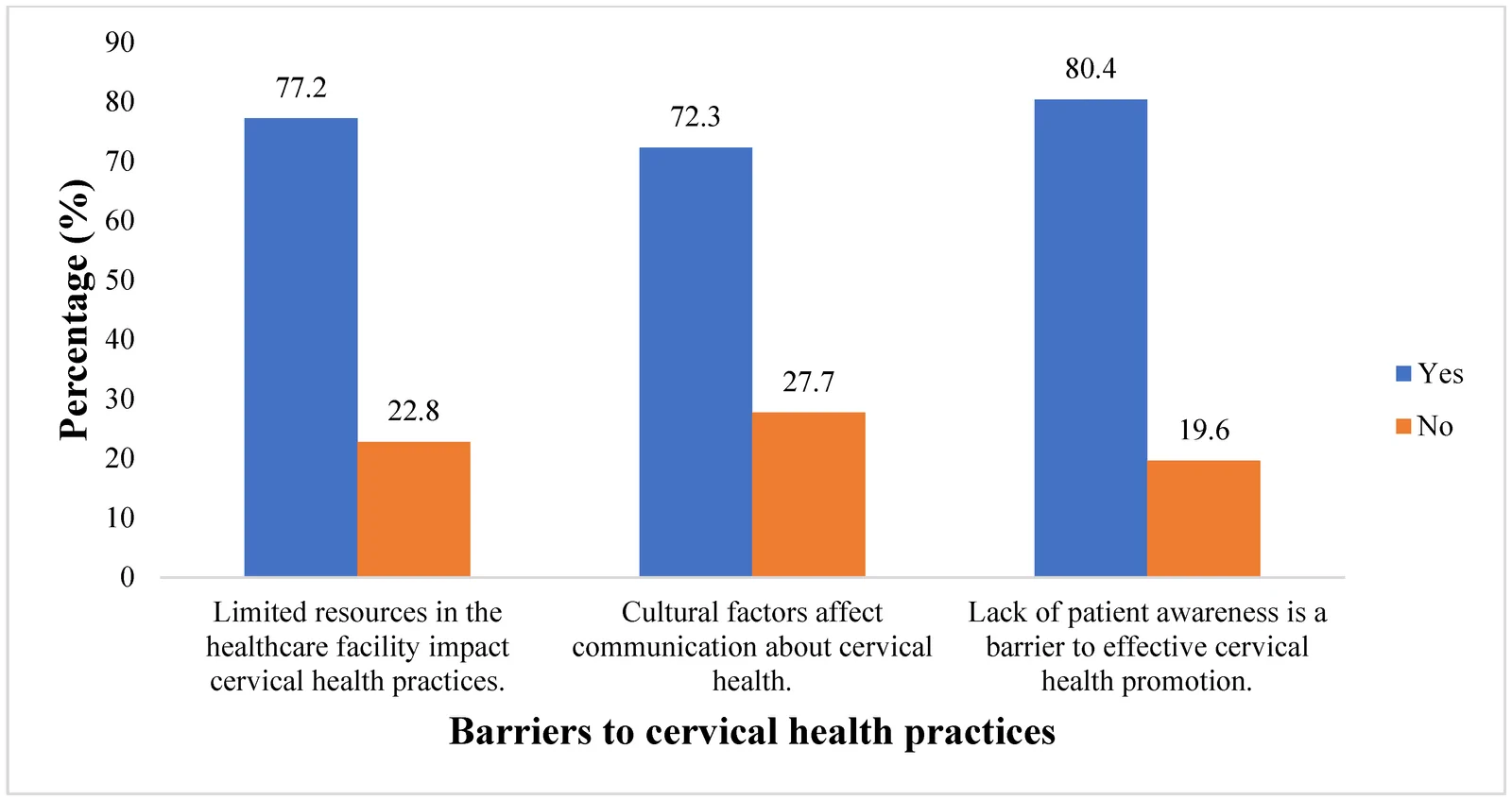
Barriers to cervical health practices.

Several barriers to cervical cancer prevention and care were reported by participating physicians. Limited resources within healthcare facilities were identified by 77.2% of respondents as an important barrier. Cultural factors were reported as influencing communication about cervical health by 72.3% of physicians, while 80.4% identified lack of patient awareness as a barrier to effective cervical cancer education and prevention (Figure 1). These findings indicate that barriers to cervical cancer prevention extend beyond individual physician knowledge and include organizational, cultural, and patient-level factors. Addressing these barriers will therefore require interventions at multiple levels of the healthcare system.

## 4. Discussion

Cervical cancer continues to be a major public health concern worldwide, especially in low- and middle-income countries such as Pakistan. The current study was conducted to assess the cervical cancer-related KAP among physicians, recognizing their pivotal role in the early detection and treatment of cases. By examining physicians’ perspectives, this study aimed to contribute valuable insights into the current state of cervical health knowledge and healthcare delivery in Lahore, Pakistan. The current study’s analysis of knowledge scores shows notable disparities based on demographic factors. Regarding gender, males had a mean score of 7.50 (SD = 0.95), while females had a slightly higher mean score of 7.61 (SD = 1.05), though this difference was not statistically significant (*p* = 0.141). While the gender-based difference in knowledge scores was not statistically significant, females scored marginally higher than males. This suggests that both genders have comparable knowledge levels, which could inform gender-neutral training interventions. In comparison, a study by Oche et al. reported a higher mean knowledge score [37]. While another study by Jiang-Li Di et al. also found a higher mean knowledge score [38]. Age emerged as a significant factor influencing knowledge levels, with younger physicians aged 26–33 years demonstrating the highest scores. The difference in knowledge across age groups was statistically significant (*p* < 0.001). This trend may be attributed to the incorporation of updated cervical cancer education in recent medical curricula, highlighting the importance of continuous professional development to ensure that knowledge remains relevant for physicians of all ages. Supporting these findings, a study by Jiang-Li Di et al. reported that younger workers (<45 years) exhibited significantly higher levels of understanding of positive results compared to their older counterparts (*p* < 0.05), further emphasizing the impact of age on knowledge acquisition and retention [38]. In the current study, higher education levels, particularly among those with MBBS and FCPS qualifications, correlated with better knowledge (*p* = 0.004). This trend highlights the importance of advanced medical training in enhancing awareness and comprehension of cervical health. Consistent with these findings, a study by Jiang-Li Di et al. reported that workers with higher education levels demonstrated significantly higher knowledge rates and scores [38]. However, direct comparison of absolute knowledge scores across studies should be made cautiously because the studies may differ in questionnaire content, number of knowledge items, scoring procedures, participant characteristics, and the specific aspects of cervical cancer knowledge assessed. Therefore, the higher or lower scores reported in previous studies should be considered contextual rather than directly comparable with the 0–9 knowledge score used in the present study.

Regarding participants’ attitudes, 170 (41.7%) participants in this study agreed that cervical cancer is widely prevalent and constitutes a significant cause of mortality among all cancers. This contrasts with findings from a study conducted in Saudi Arabia where only 52 (13.2%) participants agreed that cervical cancer is widely prevalent and a significant cause of mortality [7]. This finding represents physicians’ perceptions regarding the burden of cervical cancer and should be interpreted as an attitude-domain response rather than as a direct measure of factual knowledge. Differences from findings reported in studies from other countries may reflect variation in the wording of attitude items, study populations, cultural context, and respondents’ perceptions of cervical cancer. Consistent with findings of the current study, another study conducted by Abebaw et al. found that 151 (37.3%) participants agreed that cervical cancer is widely prevalent and a significant cause of mortality [28]. In the current study, 142 (34.8%) participants expressed a positive attitude towards routine cervical cancer screening. On the other hand, 40 (9.8%) participants held a negative view, indicating reluctance or disagreement with the screening practice. In contrast, findings from another study revealed a notable disparity in attitudes toward cervical cancer screening. Specifically, approximately one-third of participants (30.7%) viewed screening favorably. On the other hand, a significant majority (69.3%) expressed negative attitudes, reflecting reluctance towards undergoing screening [28]. Interestingly, findings from another study focusing on health workers demonstrated a positive attitude towards cervical cancer screening, with 85.47% of health workers expressing favorable views [39]. Another study observed a positive attitude towards screening in an even higher proportion of workers, with 260 (93.9%) expressing favorable views. These findings were consistent with the findings of another study conducted in Ethiopia which reported that 97% of professionals showed positive attitudes toward cervical cancer screening [33]. The responses concerning routine cervical cancer screening reflected physicians’ attitudes toward screening rather than their factual knowledge of screening recommendations.

With regard to participants’ practices the current study examined the practices of physicians regarding the recommendation of Pap smear tests for cervical cancer screening. It was found that 207 (50.7%) very frequently and 118 (28.9%) frequently advised their patients to undergo Pap smear testing for detection of cervical cancer. However, 22 practitioners (5.3%) never recommend Pap smear tests, highlighting a gap that needs to be addressed to ensure more consistent screening practices. In comparison, another study by Swapnajaswanth et al. 2014 [40] showed a more balanced split among healthcare providers regarding Pap smear test recommendations. In this study, 30 (52.6%) practitioners recommended the test, while 27 (47.4%) did not, resulting in nearly equal proportions of practitioners advising for and against the screening [40]. The notable differences between the two studies can be attributed to several factors, including variations in training, awareness, and attitudes towards cervical cancer screening guidelines among healthcare providers. In our study, the high percentage of practitioners recommending Pap smear tests suggests a strong consensus on the importance of early detection and prevention of cervical cancer. These differences underscore the importance of standardized guidelines and continuous professional education to ensure consistent and evidence-based practices in cervical cancer screening. By addressing these discrepancies and providing clear recommendations, the effectiveness of screening programs can be improved, enhancing the overall quality of patient care. Additionally, the current study examined the recommendation practices of physicians regarding the HPV vaccination. The findings revealed that 154 participants (37.7%) frequently recommended the HPV vaccine. good practices were influenced by education (*p* < 0.001), and healthcare setting (*p* < 0.01) in the adjusted analysis. In comparison, another study conducted in India by Chawla et al. found that 47% of respondents recommended that young women get vaccinated against HPV [41]. Although this percentage is higher than that obtained in our study, it still indicates that more than half of the practitioners in that study did not frequently recommend the vaccine. This highlights a gap in healthcare practices concerning HPV vaccination. Consistent with the findings of our study, another study reported that only 33.4% of physicians recommended the HPV vaccine to their patients [20]. This similarity underscores a common trend among physicians where a significant portion are not actively recommending the HPV vaccine despite its known benefits in preventing HPV-related cancers.

Overall, the present findings suggest that improving cervical cancer prevention among physicians may require more than increasing factual knowledge. The combination of lower knowledge among older and highly experienced physicians, poor practice in particular workplace categories, and less consistent reporting of counseling, documentation, follow-up, and vaccination recommendation indicates that educational interventions may need to be accompanied by organizational support. Continuing professional education, standardized preventive-care protocols, screening reminders, documentation systems, clear referral pathways, and improved access to vaccination and screening services may help address barriers identified in this study.

### 4.1. Limitations

This study has several limitations that should be considered when interpreting the findings. First, the cross-sectional design limits the ability to establish temporal or causal relationships between physicians’ demographic and professional characteristics and their knowledge, attitudes, or practices regarding cervical cancer. The observed associations should therefore be interpreted as descriptive rather than causal. Second, participants were recruited using snowball sampling from only two tertiary-care healthcare facilities in Lahore. This non-probability sampling approach may have introduced selection bias, as physicians who participated may differ systematically from those who were not reached or chose not to participate. Consequently, the findings may not be fully generalizable to physicians working in primary or secondary healthcare facilities, rural areas, or other regions of Pakistan. Third, the distribution of participants across some demographic and professional categories was uneven. In particular, relatively small numbers of physicians were represented in some age, qualification, and healthcare-facility subgroups, including those working in hospice-care facilities. These small subgroup sizes may have reduced the precision of statistical estimates and should be considered when interpreting subgroup comparisons and regression results. Fourth, the study relied on a self-administered questionnaire and self-reported practices. Although the questionnaire demonstrated modest internal consistency for the knowledge domain and higher internal consistency for the attitude and practice domains, together with evidence of content and face validity, the findings should be interpreted with consideration of the measurement characteristics of the individual domains. Self-reported responses may be affected by recall and social-desirability bias. The reported practices therefore may not necessarily reflect physicians’ actual clinical behavior. Fifth, clinical practices were not directly observed or verified through medical records. Specifically, the study did not independently verify physicians’ screening recommendations, HPV vaccination counseling, documentation of screening, referral practices, or follow-up activities. Accordingly, the practice findings should be interpreted as measures of reported behavior rather than objectively measured clinical performance. Sixth, the study focused on physicians’ KAP and perceived barriers and did not evaluate patient-level outcomes. Patient screening uptake, screening accuracy, HPV testing infrastructure, completion of referrals, treatment initiation, and cervical cancer-related clinical outcomes were not assessed. Therefore, the extent to which the reported physician practices translate into improved cervical cancer prevention or patient outcomes cannot be determined from this study. Finally, the study did not capture the professional roles or clinical responsibilities of younger physicians in sufficient detail. Because the sample contained a relatively large proportion of younger participants, differences in clinical exposure or responsibility may have contributed to observed age- and experience-related patterns. These factors should therefore be considered when interpreting the associations between age, professional experience, and cervical cancer-related KAP.

### 4.2. Policy and Future Implications

The findings of this study support the need for structured and continuous professional education in cervical cancer prevention and screening. Educational interventions should emphasize current evidence-based recommendations regarding HPV vaccination, screening, referral, follow-up, and patient counseling. Particular attention may be given to professional groups and healthcare settings in which lower knowledge or less consistent preventive practices are observed. Healthcare institutions should also strengthen organizational support for cervical cancer prevention. Standardized clinical protocols, screening reminders, documentation systems, clear referral pathways, and access to appropriate diagnostic services may improve continuity of care. Increasing the availability and affordability of HPV vaccination and cervical cancer screening is also important, particularly in resource-limited settings.

The barriers reported by physicians indicate that patient education should be culturally appropriate and accessible. Public awareness initiatives addressing misconceptions, stigma, and limited understanding of HPV and cervical cancer may improve demand for preventive services. Future studies should include larger and more diverse physician populations and use probability-based sampling where feasible. Multicenter studies involving primary, secondary, and tertiary healthcare facilities would provide a broader assessment of physician KAP across Pakistan. Longitudinal or intervention-based studies could evaluate whether continuing professional education, standardized guidelines, and institutional quality-improvement strategies lead to measurable improvements in cervical cancer prevention practices. Where feasible, future research should also incorporate objective measures such as medical-record audits or observed practice rather than relying exclusively on self-reported behavior.

## 5. Conclusions

This study demonstrated significant variation in physicians’ cervical cancer-related knowledge, attitudes and self-reported practices according to age, educational qualification, professional experience, and healthcare facility type, while knowledge did not differ significantly by sex. Knowledge scores were highest among physicians aged 26–33 years and those with MBBS and FCPS qualifications, whereas physicians with more than 20 years of experience and those working in other healthcare settings had comparatively lower scores. Regarding attitudes, physicians generally recognized the importance of cervical cancer prevention and early detection; however, confidence in discussing cervical cancer with patients may require further strengthening. Although 56.9% of physicians were classified as having good overall practices, significant differences in practice were observed across sex, age, education, professional experience, and healthcare facility type. In multivariable analysis, educational qualification and healthcare facility type remained independently associated with good practice, whereas the associations with sex, age, and professional experience were no longer statistically significant after adjustment. In addition, limited healthcare resources, cultural factors, and inadequate patient awareness were frequently reported barriers. These findings suggest that improving cervical cancer prevention requires interventions beyond individual physician knowledge, including targeted continuing professional education, standardized screening and vaccination guidance, stronger documentation and referral systems, adequate institutional resources, and culturally appropriate patient education. Future multicenter studies using objective measures of clinical practice are warranted to determine whether such interventions improve the consistency and quality of cervical cancer prevention and screening.

## Figures and Tables

**Table 2:** Mean knowledge scores of physicians.

Demographic	Variables	Mean ± SD	*p*-Value
What is your gender?	Male	7.50 (0.95)	0.141 ^a^
	Female	7.61 (1.05)	
What is your age?	18–25	7.35 (0.92)	<0.001 ***^b^
	26–33	7.90 (0.89)	
	34–41	7.48 (1.16)	
	42–49	7.33 (1.19)	
	50 and above	7.18 (1.18)	
What is your education?	MBBS	7.62 (0.92)	0.004 **^b^
	MBBS and FCPS	7.88 (0.93)	
	MD	7.46 (1.27)	
	MD and FCPS	7.22 (1.25)	
	Other	7.24 (1.04)	
How many years of experience in the healthcare?	1–5 years	7.58 (0.94)	0.010 *^b^
	5–10 years	7.60 (1.01)	
	11–20 years	7.85 (1.05)	
	More than 20 years	7.10 (1.15)	
Healthcare facility type	Hospital	7.72 (0.90)	<0.001 ***^b^
	Clinic	7.70 (0.97)	
	Hospice care facility	7.12 (0.35)	
	Other	6.90 (1.18)	

* *p* < 0.05; ** *p* < 0.01; *** *p* < 0.001. SD = Standard Deviation; ^a^ = Mann–Whitney U Test; ^b^ = Kruskal–Wallis test. MBBS = Bachelor of Medicine and Bachelor of Surgery; FCPS = Fellowship of the College of Physicians and Surgeons; MD = Doctor of Medicine.

**Table 4:** Physicians’ attitudes toward cervical cancer.

Questions	Variables	Frequency	Percentage (%)
Carcinoma of the cervix is highly prevalent and is a leading cause of deaths amongst all malignancies.	Strongly agree	62	15.2
	Agree	170	41.7
	Neutral	71	17.4
	Disagree	45	11.0
	Strongly Disagree	60	14.7
Any young woman can acquire cervical carcinoma?	Strongly Agree	67	16.4
	Agree	142	34.8
	Neutral	67	16.4
	Disagree	80	19.6
	Strongly disagree	52	12.7
Carcinoma of the cervix cannot be transmitted from one person to another?	Strongly Agree	63	15.4
	Agree	128	31.4
	Neutral	88	21.6
	Disagree	88	21.6
	Strongly Disagree	41	10.0
I consider early detection of cervical cancer a priority in patient care.	Strongly Agree	137	33.6
	Agree	134	32.8
	Neutral	78	19.1
	Disagree	29	7.1
	Strongly Disagree	30	7.4
I ask routinely to patients for screening cervical cancer.	Strongly Agree	78	19.1
	Agree	142	34.8
	Neutral	92	22.5
	Disagree	56	13.7
	Strongly Disagree	40	9.8
I feel very prepared to talk to patients about cervical cancer.	Strongly Agree	73	17.9
	Agree	110	27.0
	Neutral	132	32.4
	Disagree	63	15.4
	Strongly Disagree	30	7.4

**Table 5:** Physicians’ practices toward cervical cancer.

Questions	Variables	Frequency	Percentage (%)
I advise my patients to screen for cervical cancer.	Very Frequently	69	16.9
	Frequently	156	38.2
	Occasionally	120	29.4
	Rarely	35	8.6
	Never	28	6.9
I provide information and counselling to patients about cervical health.	Very Frequently	109	26.7
	Frequently	164	40.2
	Occasionally	61	15.0
	Rarely	36	8.8
	Never	38	9.3
Ensuring proper documentation and follow up for cervical screenings.	Very Frequently	47	11.5
	Frequently	117	28.7
	Occasionally	124	30.4
	Rarely	71	17.4
	Never	49	12.0
Comfortable to provide cervical cancer screening knowledge.	Very Frequently	61	15.0
	Frequently	128	31.4
	Occasionally	107	26.2
	Rarely	70	17.2
	Never	42	10.3
Would recommend HPV vaccination to females.	Very Frequently	78	19.1
	Frequently	154	37.7
	Occasionally	101	24.8
	Rarely	46	11.3
	Never	29	7.1
I advise my patients to undergo a Pap smear test for the detection of cervical cancer.	Very Frequently	207	50.7
	Frequently	118	28.9
	Occasionally	34	8.4
	Rarely	27	6.7
	Never	22	5.3

**Table 6:** Practices of physicians regarding cervical cancer.

Demographic Characteristics	Variables	Good Practice	Poor Practice	*p*-Value
What is your gender?	Male (*n* = 195)	122 (62.6%)	73 (37.4%)	0.026 *
	Female (*n* = 213)	110 (51.6%)	103 (48.4%)	
What is your age?	18–25 years (*n* = 158)	96 (60.8%)	62 (39.2%)	<0.001 ***
	26–33 years (*n* = 154)	101 (65.6%)	53 (34.4%)	
	34–41 years (*n* = 41)	24 (58.5%)	17 (41.5%)	
	42–49 years (*n* = 33)	6 (18.2%)	27 (81.8%)	
	50 years and above (*n* = 22)	5 (22.7%)	17 (77.3%)	
What is your education?	MBBS (*n* = 237)	169 (71.3%)	68 (28.7%)	<0.001 ***
	MBBS and FCPS (*n* = 53)	29 (54.7%)	24 (45.3%)	
	MD (*n* = 26)	8 (30.8%)	18 (69.2%)	
	MD and FCPS (*n* = 27)	6 (22.2%)	21 (77.8%)	
	Other (*n* = 65)	20 (30.8%)	45 (69.2%)	
How many years of experience in the healthcare?	1–5 years (*n* = 223)	145 (65.0%)	78 (35.0%)	<0.001 ***
	5–10 years (*n* = 99)	61 (61.6%)	38 (38.4%)	
	11–20 years (*n* = 40)	16 (40.0%)	24 (60.0%)	
	More than 20 years (*n* = 46)	10 (21.7%)	36 (78.3%)	
Healthcare facility type	Hospital (*n* = 291)	186 (63.9%)	105 (36.1%)	<0.001 ***
	Clinic (*n* = 34)	23 (67.6%)	11 (32.4%)	
	Hospice care facility (*n* = 8)	5 (62.5%)	3 (37.5%)	
	Other (*n* = 75)	18 (24.0%)	57 (76.0%)	

* *p* < 0.05; *** *p* < 0.001. MBBS = Bachelor of Medicine and Bachelor of Surgery; FCPS = Fellowship of the College of Physicians and Surgeons; MD = Doctor of Medicine.

**Table 7:** Regression analysis of good practices related to cervical cancer.

Demographic Characteristics	Variables	OR	95% CI		*p*-Value	ORa	95% CI		*p*-Value
			Lower	Upper			Lower	Upper	
Gender	Male	0.693	0.430	0.949	0.026 *	0.974	0.620	1.531	0.911
	Female	Reference							
Age	18–25	0.190	0.067	0.541	0.002 **	1.432	0.315	6.514	0.642
	26–33	0.154	0.054	0.442	<0.001 ***	1.093	0.245	4.875	0.907
	34–41	0.208	0.064	0.675	0.009 **	0.482	0.120	1.941	0.304
	42–49	1.324	0.349	5.019	0.680	2.170	0.491	9.589	0.307
	50 and above	Reference							
Education	MBBS	0.179	0.098	0.325	<0.001 ***	0.240	0.116	0.496	<0.001 ***
	MBBS and FCPS	0.368	0.173	0.783	0.009 **	0.599	0.239	1.502	0.275
	MD	1.000	0.373	2.679	1.000	1.395	0.455	4.279	0.560
	MD and FCPS	1.556	0.545	4.441	0.409	1.171	0.339	4.041	0.803
	Other	Reference							
Years of experience	1–5 years	0.149	0.070	0.317	<0.001 ***	0.770	0.206	2.870	0.696
	5–10 years	0.173	0.077	0.389	<0.001 ***	0.726	0.197	2.673	0.630
	11–20 years	0.417	0.162	1.071	0.069	0.766	0.233	2.527	0.662
	More than 20 years	Reference							
Healthcare facility type	Hospital	0.178	0.100	0.319	<0.001 ***	0.376	0.175	0.808	0.012
	Clinic	0.151	0.062	0.369	<0.001 ***	0.199	0.068	0.580	0.003 **
	Hospice care facility	0.189	0.041`	0.872	0.033 *	0.228	0.042	1.243	0.088
	Other	Reference							

* *p* < 0.05; ** *p* < 0.01; *** *p* < 0.001. OR = Odds Ratio; ORa = Adjusted Odds Ratio; CI = Confidence Interval; MBBS = Bachelor of Medicine and Bachelor of Surgery; FCPS = Fellowship of the College of Physicians and Surgeons; MD = Doctor of Medicine.

## Data Availability

The datasets generated and/or analyzed during the current study are available from the corresponding author upon reasonable request. The data are not publicly available because they contain potentially sensitive information related to participants’ responses and professional practices, and public disclosure could compromise participant confidentiality.

## Supplementary Materials

The following supporting information can be downloaded at: https://www.mdpi.com/article/10.3390/healthcare14213613/s1, File S1: Questionnaire.

## References

[1] Singh D., Vignat J., Lorenzoni V., Eslahi M., Ginsburg O., Lauby-Secretan B., Arbyn M., Basu P., Bray F., Vaccarella S. (2023). Global estimates of incidence and mortality of cervical cancer in 2020: A baseline analysis of the WHO Global Cervical Cancer Elimination Initiative. Lancet Glob. Health.

[2] Li Y., Song W., Gao P., Guan X., Wang B., Zhang L., Yao Y., Guo Y., Wang Y., Jiang S. (2025). Global, regional, and national burden of breast, cervical, uterine, and ovarian cancer and their risk factors among women from 1990 to 2021, and projections to 2050: Findings from the global burden of disease study 2021. BMC Cancer.

[3] Singh G.K., Azuine R.E., Siahpush M. (2012). Global Inequalities in Cervical Cancer Incidence and Mortality are Linked to Deprivation, Low Socioeconomic Status, and Human Development. Int. J. Matern. Child Health AIDS.

[4] Hull R., Mbele M., Makhafola T., Hicks C., Wang S., Reis R.M., Mehrotra R., Mkhize-Kwitshana Z., Kibiki G., Bates D.O. (2020). Cervical cancer in low and middle-income countries (Review). Oncol. Lett..

[5] LaVigne A.W., Triedman S.A., Randall T.C., Trimble E.L., Viswanathan A.N. (2017). Cervical cancer in low and middle income countries: Addressing barriers to radiotherapy delivery. Gynecol. Oncol. Rep..

[6] World Health Organization (2021). WHO Guideline for Screening and Treatment of Cervical Pre-Cancer Lesions for Cervical Cancer Prevention.

[7] Heena H., Durrani S., AlFayyad I., Riaz M., Tabasim R., Parvez G., Abu-Shaheen A. (2019). Knowledge, Attitudes, and practices towards cervical cancer and screening amongst female healthcare professionals: A cross-sectional study. J. Oncol..

[8] Chughtai N., Perveen K., Gillani S.R., Abbas A., Chunara R., Manji A.A., Karani S., Noorali A.A., Zakaria M., Shamsi U. (2023). National cervical cancer burden estimation through systematic review and analysis of publicly available data in Pakistan. BMC Public Health.

[9] Zhou Q., Chen P., Guo Y., Song Y.-F. (2025). The Evolving Landscape of Cervical Cancer Histology: A Comprehensive Population-Based Analysis of Global Patterns and Trends in 2022. SSRN.

[10] Williamson A.-L. (2023). Recent Developments in Human Papillomavirus (HPV) Vaccinology. Viruses.

[11] Aziz H., Mehmood H., Arif S., Afzal K., Hanif S., Saeed M.A., Faheem M. (2025). Distribution of HPV Types in Cervical Cancer in Pakistan: Implications for Screening and Vaccination Programs. J. Clin. Lab. Anal..

[12] Hemalatha T., Rehman W., Idrees S., Ghailan K., Azeez F. (2018). Knowledge, Attitude and Preventive Practices among the Female Students Regarding Cervical Cancer Screening and HPV Vaccination in Jazan University. J. Women’s Health.

[13] Burd E.M. (2003). Human papillomavirus and cervical cancer. Clin. Microbiol. Rev..

[14] Chrysostomou A.C., Stylianou D.C., Constantinidou A., Kostrikis L.G. (2018). Cervical cancer screening programs in Europe: The transition towards HPV vaccination and population-based HPV testing. Viruses.

[15] Wudtisan J., Tantipalakorn C., Charoenkwan K., Sreshthaputra R., Srisomboon J. (2019). Factors Associated with Development of High-Grade Squamous Intraepithelial Lesions of the Uterine Cervix in Women Younger than 30 Years. Asian Pac. J. Cancer Prev..

[16] Desaraju A., Kodey P.D. (2021). Knowledge, attitude, and practices of cervical cancer screening in health care providers in a teaching hospital. J. Dr. NTR Univ. Health Sci..

[17] Mwaka A.D., Okello E.S., Wabinga H., Walter F.M. (2015). Symptomatic presentation with cervical cancer in Uganda: A qualitative study assessing the pathways to diagnosis in a low-income country. BMC Women’s Health.

[18] Obol J.H., Lin S., Obwolo M.J., Harrison R., Richmond R. (2021). Knowledge, attitudes, and practice of cervical cancer prevention among health workers in rural health centres of Northern Uganda. BMC Cancer.

[19] El-Zein M., Richardson L., Franco E.L. (2016). Cervical cancer screening of HPV vaccinated populations: Cytology, molecular testing, both or none. J. Clin. Virol..

[20] Narayana G., Suchitra J., Suma G.K., Deepthi G., Jyothi C.D., Kumar B.P. (2020). Physician’s knowledge, attitude, and practice towards Human Papilloma Virus (HPV) vaccine recommendation in anantapur district, Andhra Pradesh, India. Arch. Pharm. Pract..

[21] Ngambi D., Ramathuba D.U. (2024). Challenges regarding the implementation of cervical cancer screening guidelines in Limpopo province, South Africa. Afr. J. Prim. Health Care Fam. Med..

[22] Mansour R., Abdel-Razeq H., Al-Hussaini M., Shamieh O., Al-Ibraheem A., Al-Omari A., Mansour A.H. (2024). Systemic Barriers to Optimal Cancer Care in Resource-Limited Countries: Jordanian Healthcare as an Example. Cancers.

[23] Maseko F.C., Chirwa M.L., Muula A.S. (2015). Health systems challenges in cervical cancer prevention program in Malawi. Glob. Health Action.

[24] Alodhialah A.M., Almutairi A.A., Almutairi M. (2024). Assessing Barriers to Cancer Screening and Early Detection in Older Adults in Saudi Arabia: A Mixed-Methods Approach to Oncology Nursing Practice Implications. Curr. Oncol..

[25] Rathod S., Gupta A., Sethi N., Dande A., Potdar J. (2023). Empowering Women’s Health: Insights Into HPV Vaccination and the Prevention of Invasive Cervical Cancer. Cureus.

[26] Chauhan M., Gupta S., Gandhi R. (2020). Study of knowledge, attitude, and practice regarding cervical cancer and its screening among health service providers of Udaipur. Int. J. Clin. Obstet. Gynaecol..

[27] Easwaran V., Shorog E.M., Alshahrani A.A., Mohammad A.A.S., Sadiq M.M.J., Alavudeen S.S., Khan N.A., Akhtar S., Almeleebia T.M., Alshahrani S.M. (2023). Knowledge, Attitudes, and Practices Related to Cervical Cancer Prevention and Screening among Female Pharmacy Students at a Public University in a Southern Region of Saudi Arabia. Healthcare.

[28] Abebaw E., Tesfa M., Gezimu W., Bekele F., Duguma A. (2022). Female healthcare providers’ knowledge, attitude, and practice towards cervical cancer screening and associated factors in public hospitals of Northwest Ethiopia. SAGE Open Med..

[29] Shah V., Vyas S., Singh A., Shrivastava M. (2012). Awareness and knowledge of cervical cancer and its prevention among the nursing staff of a tertiary health institute in Ahmedabad, Gujarat, India. Ecancermedicalscience.

[30] Daniel W.W. (1978). Biostatistics: A Foundation for Analysis in the Health Sciences.

[31] Pourhoseingholi M.A., Vahedi M., Rahimzadeh M. (2013). Sample size calculation in medical studies. Gastroenterol. Hepatol. Bed Bench.

[32] Sait K.H. (2011). Knowledge, attitudes, and practices regarding cervical cancer screening among physicians in the Western Region of Saudi Arabia. Saudi Med. J..

[33] Goedken J., Sharling L., Kress C.M., Desalegn D., Blumberg H.M., Owen-Smith A. (2015). Knowledge, attitudes, and practices regarding cervical cancer and screening among Ethiopian health care workers. Int. J. Women’s Health.

[34] Kulshreshtha S., Gupta G., Goyal G., Gupta K., Davda K. (2024). Item Analysis of Single Best Response Type Multiple Choice Questions for Formative Assessment in Obstetrics and Gynaecology. J. Obstet. Gynecol. India.

[35] Alajmi S.M., Alsulami T.M., Ben Mudayhish M.A., Alhawas M.A., Alangari M.S., Omair A., Ben Mudayhish M.A., Alhawas M.A., Alfarhan A.I. (2023). Knowledge and attitude of medical students towards helicobacter pylori infection and its prevention and management: A study from riyadh, Saudi Arabia. Cureus.

[36] Imam N.H. (2026). A Survey on Student Awareness of Spoofing Attacks in Saudi Arabia. Big Data Cogn. Comput..

[37] Oche M., Kaoje A., Gana G., Ango J. (2013). Cancer of the cervix and cervical screening: Current knowledge, attitude and practices of female health workers in Sokoto, Nigeria. Int. J. Med. Med. Sci..

[38] Di J.-L., Rutherford S., Wu J.-L., Song B., Ma L., Chen J.-Y., Chu C. (2016). Knowledge of cervical cancer screening among health care workers providing services across different socio-economic regions of China. Asian Pac. J. Cancer Prev..

[39] Chawla B., Taneja N., Awasthi A.A., Kaur K.N., Janardhanan R. (2021). Knowledge, attitude, and practice on screening toward cervical cancer among health professionals in India—A review. Women’s Health.

[40] Swapnajaswanth M., Suman G., Suryanarayana S., Murthy N. (2014). Perception and practices on screening and vaccination for carcinoma cervix among female healthcare professional in tertiary care hospitals in Bangalore, India. Asian Pac. J. Cancer Prev..

[41] Chawla P.C., Chawla A., Chaudhary S. (2016). Knowledge, attitude & practice on human papillomavirus vaccination: A cross-sectional study among healthcare providers. Indian J. Med. Res..

