# Supplementary material for: Assessing Physicians’ Knowledge, Attitudes, and Practices Regarding Cervical Cancer Prevention, Diagnosis, and Management in Lahore, Pakistan
Source: Healthcare (Basel). 2026 Oct 26;14(21):3613. doi: 10.3390/healthcare14213613 (PMC13762984; doi:10.3390/healthcare14213613)
Supplement: Supplementary file 1 [file healthcare-14-03613-s001.zip › healthcare-4551283-supplementary.pdf]

Assessing Physicians' Knowledge, Attitudes, and Practices Regarding Cervical Cancer Prevention, Diagnosis, and Management in Lahore, Pakistan

File S1: Questionnaire

Section S1: Demographic Information

1) What is your gender?

a) Male

b) Female

2) What is your Age?

a) 18-25

b) 26-33

c) 34-41

d) 42-49

e) 50 and above

3) What is your education?

a) MBBS

b) MBBS & FCPS

c) MD

d) MD & FCPS

e) Other

4) How many years of experience in healthcare?

a) 1-5 years

b) 5-10 years

c) 11-20 years

d) More than 20 years

5) Healthcare Facility Type:

a) Hospital

b) Clinic

c) Hospice care facility

d) Other

Section S2: Knowledge about Cervical Cancer

6) Cervical cancer, globally the most frequently observed cancer in women.

a) Yes

b) No

7) Risk factors associated with cervical cancer.

a) Multiple sexual partners

b) Early sexual intercourse

c) HPV infection

d) HIV infection

e) Smoking

f) Contraceptive methods

g) I don't know

8) Most common sign and symptoms of cervical cancer.

a) Vaginal bleeding

b) Foul-smelling vaginal discharge

c) Contact bleeding

d) Abdominal Pain

e) Without any symptom

f) I don't know

9) Cervical cancer is preventable.

a) Yes

b) No

10) What can be done to prevent cervical cancer?

a) Regular pap-smear test

b) HPV vaccine

c) Colposcopy

d) Monogamy

e) Avoiding multiple sexual partners

f) Avoiding early sexual intercourse

g) I don’t know

11) What are the methods used for screening of cervical cancer?

a) Pap smear test

b) Gynecological examination

c) HPV DNA test

d) Fluid-based cytology

e) Visual examination

f) I don’t know

12) At what age do you begin screening asymptomatic average-risk women for cervical cancer?

a) Before age 25 years

b) 25–29 years

c) 30–34 years

d) More than 35 years

e) I don’t know

13) Recommended interval for screening of cervical cancer.

a) Every 6 months

b) Every 1 year

c) Every 3 years

d) Every 5 years

e) I don’t know

14) Treatment of cervical cancer.

a) Chemotherapy

b) Radiotherapy

c) Depends on stage of disease

d) Surgery

e) I don’t know

Section S3: Attitudes towards Cervical Health

15) Carcinoma of the cervix is highly prevalent and is a leading cause of deaths amongst all malignancies.

a) Strongly Agree

b) Agree

c) Neutral

d) Disagree

e) Strongly Disagree

16) Any young woman can acquire cervical carcinoma?

a) Strongly Agree

b) Agree

c) Neutral

d) Disagree

e) Strongly Disagree

17) Carcinoma of the cervix cannot be transmitted from one person to another.

a) Strongly Agree

b) Agree

c) Neutral

d) Disagree

e) Strongly Disagree

18) I consider early detection of cervical cancer a priority in patient care.

a) Strongly Agree

b) Agree

c) Neutral

d) Disagree

e) Strongly Disagree

19) I ask routinely to patients for screening cervical cancer.

a) Strongly Agree

b) Agree

c) Neutral

d) Disagree

e) Strongly Disagree

20) I feel very prepared to talk to patients about cervical cancer.

a) Strongly Agree

b) Agree

c) Neutral

d) Disagree

e) Strongly Disagree

<u>Section S4: Practices Related to Cervical Health</u>

21) I advise my patients to screen for cervical cancer.

a) Very Frequently

b) Frequently

c) Occasionally

d) Rarely

e) Never

22) I provide information and counseling to patients about cervical health.

a) Very Frequently

b) Frequently

c) Occasionally

d) Rarely

e) Never

23) Ensuring proper documentation and follow-up for cervical screenings.

a) Very Frequently

b) Frequently

c) Occasionally

d) Rarely

e) Never

24) Comfortable to provide cervical cancer screening knowledge.

a) Very Frequently

b) Frequently

c) Occasionally

d) Rarely

e) Never

25) Would recommend HPV vaccination to females.

a) Very Frequently

b) Frequently

c) Occasionally

d) Rarely

e) Never

26) I advise my patients to undergo a Pap smear test for the detection of cervical cancer.

a) Very Frequently

b) Frequently

c) Occasionally

d) Rarely

e) Never

Section S5: Challenges and Barriers Related to Cervical Health Practices

27) Limited resources in the healthcare facility impact cervical health practices.

a) Yes

b) No

28) Cultural factors affect communication about cervical health.

a) Yes

b) No

29) Lack of patient awareness is a barrier to effective cervical health promotion.

a) Yes

b) No
